# Effectiveness of indapamide/amlodipine single-pill combination in patients with isolated systolic hypertension: post-hoc analysis of the ARBALET study

**DOI:** 10.1186/s12872-022-02514-y

**Published:** 2022-03-04

**Authors:** Zh. D. Kobalava, Eteri L. Kolesnik, E. K. Shavarova, L. A. Goreva, L. V. Karapetyan

**Affiliations:** The Peoples’ Friendship University of Russia (RUDN University), Vavilova st., 61/1, Moscow, Russia 119296

**Keywords:** Indapamide/amlodipine single-pill combination, Combination therapy, Isolated systolic hypertension

## Abstract

**Background:**

This study evaluated the effectiveness of treatment with an indapamide/amlodipine single-pill combination (SPC) in outpatients with uncontrolled isolated systolic hypertension (ISH) aged over 55 years in real-life clinical practice.

**Methods:**

This was a post-hoc analysis of the subgroup of patients with ISH from ARBALET, a 3-month, multicenter, observational, open-label study conducted in Russia among patients with grade I or II hypertension who were either uncontrolled on previous antihypertensive treatment or treatment-naïve. The effectiveness of indapamide/amlodipine SPC was assessed by the change in office systolic blood pressure (SBP) and the rate of target SBP (< 140 mmHg) achievement at 2 weeks, 1 month and 3 months, in four age groups: 55–59 years, 60–69 years, 70–79 years, and 80 years or older.

**Results:**

The ARBALET study recruited 2217 patients, of whom 626 had ISH and were included in this post-hoc analysis (mean age 66.1 ± 7.8 years; 165 men [26.4%] and 461 women [73.6%]). Target SBP < 140 mmHg was achieved in 43%, 75% and 93% of patients at 2 weeks, 1 and 3 months, respectively. SBP decreased from baseline by 18.8 ± 10.5 mmHg, 27.2 ± 10.6 mmHg and 31.8 ± 9.9 mmHg at 2 weeks, 1 month and 3 months, respectively. In the groups of patients aged 55–59, 60–69, 70–79, and ≥ 80 years, SBP reductions at 3 months compared with baseline were − 30.3 ± 9.4, − 32.4 ± 9.7, − 32.5 ± 10.7, and − 28.9 ± 9.6 mmHg, respectively.

**Conclusion:**

This post-hoc analysis of the observational ARBALET study showed that indapamide/amlodipine SPC was associated with significant reductions in BP and high rates of target BP achievement in a broad age range of patients with ISH treated in routine clinical practice.

**Study registration number:**

ISRCTN40812831.

## Introduction

Isolated systolic hypertension (ISH) is defined as a systolic blood pressure (SBP) ≥ 140 mmHg with a diastolic blood pressure (DBP) < 90 mmHg [1, 2]. There is a linear increase in both SBP and DBP up to around 45 years in men and 55 years in women after which the prevalence of ISH begins to increase [3, 4]. In the NHANES III study, the proportion of those with ISH among all hypertensive patients aged 45–54 years was 24%, among 55–64-year olds it was 47%, among 65–74-year olds it was 66%, and in over 75-year olds it was 73% [5]. ISH is associated with a two- to fourfold increase in the risk of myocardial infarction (MI), left ventricular hypertrophy (LVH), renal dysfunction, stroke, and cardiovascular death [4]. In the Physician’s Health Study of apparently healthy men aged 40–84 years, even borderline ISH significantly increased the risk of cardiovascular disease by 32%, stroke by 42%, cardiovascular death by 56% and all-cause mortality by 22% [6]. The debate on whether “to treat or not to treat hypertension, including ISH in the elderly” is therefore a thing of the past.

Hypertension in older patients is influenced by a number of features that should be considered when selecting treatment options, including increased arterial stiffness, circadian rhythm disturbances, high BP variability, and reduced plasma renin activity leading to the development of sodium-volume dependent hypertension. Furthermore, in a high proportion of patients, antihypertensive therapy is associated with suboptimal rates of target BP achievement [7].

Achieving BP control is essential for the improvement of hypertension-related outcomes, and often requires prescription of a combination of antihypertensive agents with different mechanisms of action. A single-pill combination (SPC) is the preferred method of administration as it reduces the pill burden and can improve treatment adherence [1].

In 2017, a unique SPC combining the thiazide-like diuretic indapamide sustained release at a dose of 1.5 mg with the calcium channel blocker (CCB) amlodipine at a dose of 5 or 10 mg was registered in Russia.

The ARBALET study [8, 9] was designed to evaluate the antihypertensive effectiveness and tolerability of the indapamide/amlodipine SPC in patients with hypertension over 55 years of age in real clinical practice. The results showed that 90% of patients achieved target BP level by the third month of treatment and that the number of patients with a pulse pressure (PP) < 60 mmHg increased from 7.8 to 82%. Herein, we present a post-hoc analysis of the ARBALET study whose aim was to assess the effectiveness of treatment with indapamide/amlodipine SPC specifically in outpatients with ISH.

## Methods

### Study design

ARBALET was a 3-month, multicenter, open-label, observational, uncontrolled study, conducted between November 2017 and March 2018. A total of 730 physicians from 57 regions of the Russian Federation enrolled 2217 patients who in the physician’s opinion, required an adjustment of their antihypertensive therapy, either by addition of indapamide/amlodipine SPC to previous therapy, or by replacement of the effective free combination of the same agents with the SPC. Eligible patients were aged ≥ 55 years, had primary hypertension diagnosed at least 3 months before inclusion in the study, and had uncontrolled BP on previous antihypertensive therapy (office SBP 140–179 mm Hg), or were antihypertensive treatment-naïve patients with grade I or II hypertension or with PP ≥ 60 mmHg. From this population, patients with ISH (n = 626 patients) were included in the current post-hoc analysis. Exclusion criteria were: patients having office BP ≥ 180/110 mmHg despite antihypertensive treatment (at inclusion visit) or ≥ 200/110 mmHg if antihypertensive treatment-naïve; resistant hypertension (use of 3 antihypertensive drugs of different classes at the best tolerated doses, one of which must be a diuretic); a history of myocardial infarction, unstable angina, or cerebrovascular accident within the prior 6 months; New York Heart Association (NYHA) class III or IV chronic heart failure (CHF); type 1 diabetes mellitus (DM) or decompensated type 2 DM; any severe decompensated concomitant diseases; inability to understand the nature of the program and follow the recommendations; contraindications or known intolerance to diuretics and CCB (including indapamide and amlodipine); or participation in any other clinical study within 30 days prior to the program.

Each treating physician selected three or more consecutive patients who met the above criteria.

The indapamide/amlodipine SPC dose was selected by the physician from two available options (indapamide/amlodipine 1.5/5 mg or 1.5/10 mg). In all cases, treatment was prescribed in accordance with the instructions for use of the drugs, after the patient had signed an informed consent form.

The study included three pre-scheduled patient visits at 2 weeks, 1 and 3 months after the inclusion visit. Patients were divided into four age groups: 55–59, 60–69, 70–79, and 80–90 years. At each visit, after recording the timings of previous drug intake, the physician measured BP and heart rate (HR) and completed the case report form. After 5 min of rest, office BP and heart rate (HR) were measured three times at 1- to 2-min intervals in a sitting position, on the right arm and the last two measurements were registered. In this observational study, the methods of routine clinical practice were used, including the auscultatory method for measuring BP in the physician’s office (Korotkoff technique). Ordinary mechanic tensiometers calibrated by regional metrological centers were used in the study, in accordance with routine clinical practice in Russia.

Based on the obtained data, a decision concerning treatment continuation was made by the physician and the dose of drug was titrated at the week 2 or 1-month visits, if necessary. Adverse events were monitored throughout the study.

The primary efficacy endpoints were change in SBP and DBP values at the final visit versus baseline (recorded at the inclusion visit), and rate of target BP achievement. Secondary efficacy endpoints in this subgroup of patients with ISH included the rates of target SBP (< 130 mmHg) and PP (< 60 mmHg) achievement.

### Statistical analysis

All parameters were analysed using descriptive statistics methods. Changes in mean SBP and DBP values (with corresponding 95% confidence intervals [CI]) were evaluated in the per-protocol population. To assess the differences in the normally distributed parameters, the Student’s t-test for paired measurements was used; the Wilcoxon nonparametric rank-sum test was used for parameters that were not normally distributed. The proportions (with corresponding 95% CI) of patients achieving target BP, as well as those who responded to treatment were also calculated.

### Compliance with ethics guidelines

All diagnostic procedures were performed after written informed consent had been provided by the patient. The study was conducted in accordance with the principles of Good Clinical Practice (GCP) and the Declaration of Helsinki. The study protocol was approved by the Ethical Committee for Medical Studies of the People’s Friendship University of Russia (RUDN).

## Results

### Patient demographics

The primary ARBALET study population comprised 2217 patients. Mean age was 64.2 ± 7.4 years and 692 (31.2%) were men. Mean baseline SBP was 161.7 ± 10.3 mmHg and mean DBP was 90.7 ± 9.7 mmHg. Prior to inclusion in the study, 28.0% of patients were being prescribed antihypertensive monotherapy, 38.7% were receiving two antihypertensive agents, 15.0% three agents, 4.2% four agents, and 0.5% of patients were receiving five antihypertensive agents. At study entry, 68.5% were prescribed indapamide/amlodipine SPC at a dose of 1.5/5 mg and 31.5% were prescribed a dose of 1.5/10 mg. The number of concomitant drugs taken by patients before inclusion in the study had an influence on the prescribed SPC dose. For most patients (96.5%), the SPC dose remained stable during the study. At 3 months, 60.7% of patients were receiving a dose of 1.5/5 mg, and 39.3% were receiving 1.5/10 mg. In a small number of patients some dose changes were noted, which mainly consisted of a dose increase at the final visit in 2.0% of patients.

The remainder of this paper focuses on the post-hoc analysis population of 626 patients in the ARBALET study who presented with ISH. Baseline patient characteristics in the ISH population were similar to those of the main population [8] except for a greater proportion of patients aged 70–79 years of age (27.3% vs 19.6%) and a higher mean age (66.1 ± 7.8 vs 64.2 ± 7.4 years). Among the ISH population there were 165 (26.4%) men and 461 (73.6%) women. The main clinical and demographic characteristics of the ISH patients are presented in Table 1. Mean baseline SBP was 159.2 ± 8.7 mmHg, DBP was 79.7 ± 6.5 mmHg, PP 79.5 ± 10.7 mmHg, and HR 71.7 ± 8.0 beats per minute. Analysis of cardiovascular risk factors showed that more than two-thirds of patients had dyslipidemia (n = 434 patients; 69.3%) and more than half had abdominal obesity (n = 348; 55.6%). The most prevalent concomitant diseases/conditions were echocardiographically-confirmed LVH (n = 455; 72.7%), CAD (n = 208; 33.2%), and CHF (n = 276; 44.1%).

**Table 1:** Baseline characteristics of patients with ISH (n = 626)

Parameter	Value
Men, n (%)	165 (26.4)
Women, n (%)	461 (73.6)
Age, years, mean ± SD	66.1 ± 7.8
55–59 years, n (%)	137 (21.9)
60–69 years, n (%)	281 (44.9)
70–79 years, n (%)	171 (27.3)
≥ 80 years, n (%)	30 (4.8)
*Risk factors*	
Current smoker, n (%)	103 (16.5)
Dyslipidemia, n (%)	434 (69.3)
Fasting plasma glucose ≥ 5.6 mmol/L, n (%)	146 (23.3)
Abdominal obesity, n (%)	348 (55.6)
Family history of CVD, n (%)	160 (25.6)
*Concomitant diseases and conditions*	
Confirmed LVH, n (%)	455 (72.7)
Proteinuria, n (%)	34 (5.4)
CAD, n (%)	208 (33.2)
Stable angina, n (%)	138 (22.0)
History of myocardial infarction, n (%)	40 (6.4)
History of coronary revascularisation, n (%)	29 (4.6)
History of stroke or TIA, n (%)	23 (3.7)
Peripheral artery disease, n (%)	83 (13.3)
Class I or II CHF, n (%)	276 (44.1)
Type 2 diabetes mellitus, n (%)	72 (11.5)
COPD/asthma, n (%)	37 (5.9)

*CAD* coronary artery disease, *CHF* chronic heart failure, *CVD* cardiovascular disease, *COPD* chronic obstructive pulmonary disease, *LVH* left ventricular hypertrophy, *ISH* isolated systolic hypertension, *SD* standard deviation, *TIA* transient ischemic attack

At baseline, all participants were prescribed an indapamide/amlodipine SPC, which replaced previous antihypertensive treatment in 460 (73.5%) patients, was added to a current antihypertensive regimen in 80 (12.8%) patients, and was initiated in 86 (13.7%) treatment-naïve patients with ISH. The 1.5/5 mg dose was prescribed in 466 (74.4%) patients and the 1.5/10 mg dose in 160 (25.6%).

### Concomitant treatments

A total of 540 out of 626 patients (86.3%) had received previous antihypertensive treatment and 86 were treatment naïve. Previous antihypertensive agents included angiotensin-converting enzyme (ACE) inhibitors, angiotensin II receptor blockers (ARB), beta-blockers, CCB, diuretics, and imidazoline receptor agonists. Of the 540 on previous antihypertensive treatment 175 (32.4%) were receiving monotherapy and 365 (67.6%) were receiving combination therapy prescribed as either a free combination (n = 305; 83.6%) or as a SPC (n = 60; 16.4%).

A proportion of patients received concomitant antihypertensive therapy during the study in addition to the indapamide/amlodipine SPC including ACE inhibitors in 174 patients (27.8%), ARB in 102 (16.3%), beta-blockers in 210 (33.6%), CCB in 3 (0.5%), diuretics in 12 (1.9%), and imidazoline receptor agonists in 10 (1.6%) patients.

### Changes in SBP levels

**Fig. 1:**
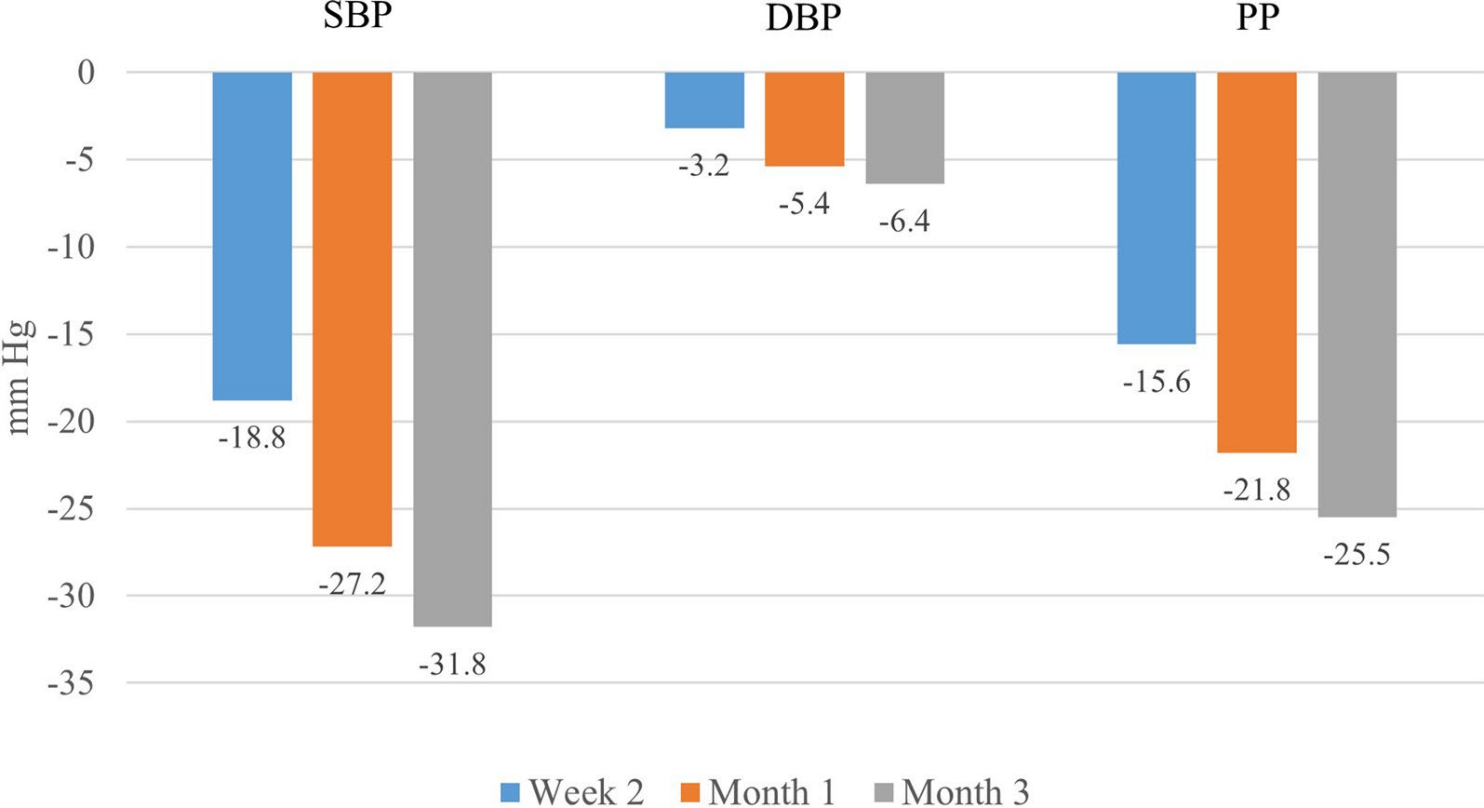
Changes in blood pressure (BP) during the study

**Fig. 2:**
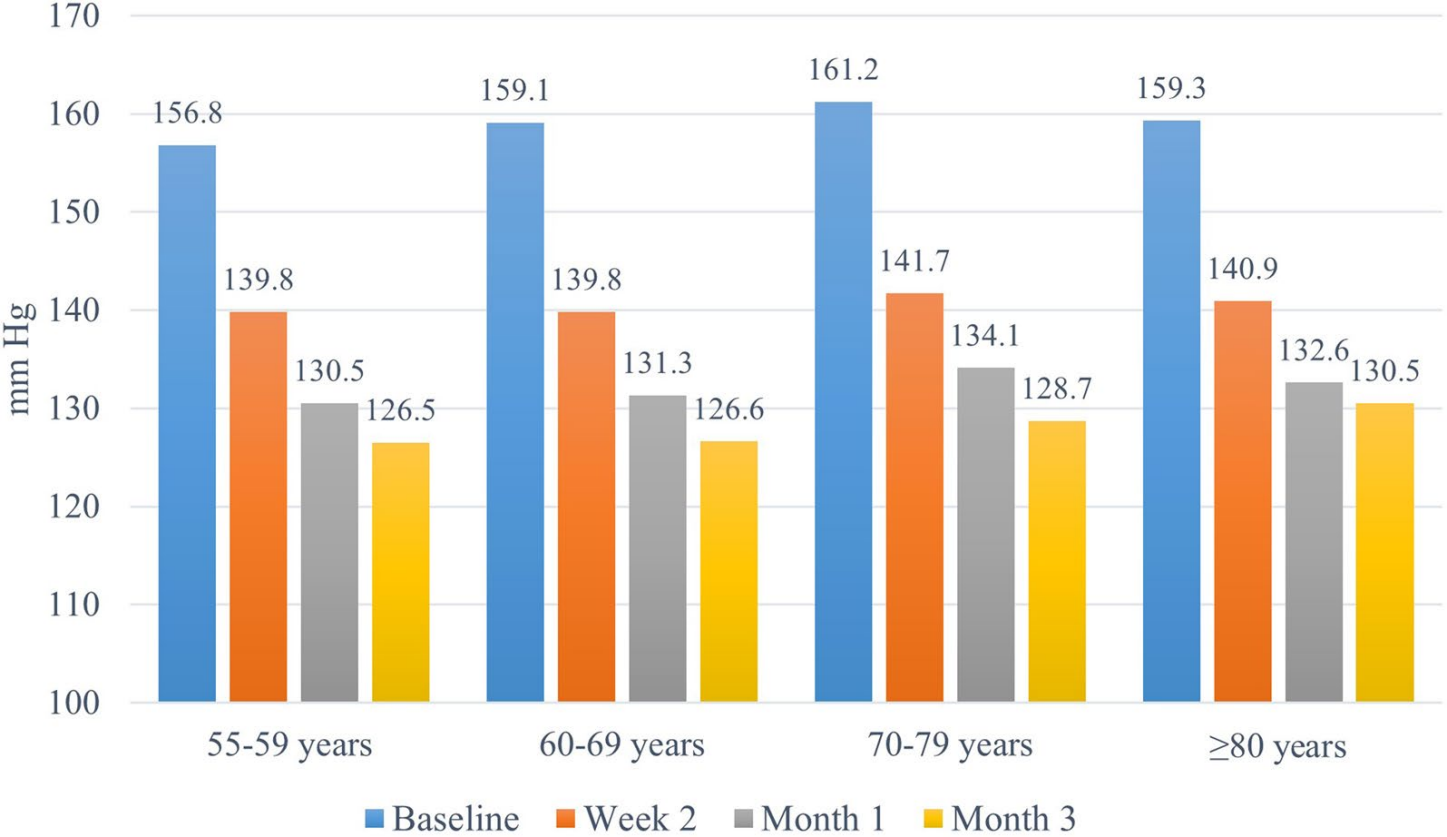
Changes in mean systolic blood pressure (SBP) during the treatment in different age groups

BP measurements were available for 615 (98.2%) patients who completed the study in accordance with the study protocol. Statistically significant reductions compared with baseline were observed for SBP, DBP and PP from Week 2 and remained significant for the duration of the study (Fig. 1). Changes in SBP levels by age group are presented in Fig. 2. After 3 months of treatment with the indapamide/amlodipine SPC, significant SBP decreases from baseline were observed in each age group: − 30.3 ± 9.4 mmHg (from 156.8 ± 8.4 to 126.5 ± 7.3), − 32.4 ± 9.7 mmHg (from 159.1 ± 8.5 to 126.6 ± 7.1), − 32.5 ± 10.7 mmHg (from 161.2 ± 8.9 to 128.7 ± 7.7), and − 28.9 ± 9.6 mmHg (from 159.3 ± 8.5 to 130.5 ± 7.1) in the 55–59, 60–69, 70–79, and 80 years and older age groups, respectively.

### Rate of target SBP achievement

SBP reductions to less than 140 mmHg were observed after 2 weeks in 265 (43.1%) patients, after 1 month in 458 (74.5%) patients, and after 3 months in 569 (92.5%) patients. An SBP level of less than 130 mmHg was achieved after 2 weeks in 74 (12.0%) patients, after 1 month in 209 (34.0%) patients and after 3 months in 344 (55.9%) patients.

Patients aged 55–59 years old had the highest levels of target SBP level achievement after 3 months of treatment (95% achieved a target of < 140 mmHg and 61% a target of < 130 mmHg), while the lowest proportions of patients achieving the two blood pressure targets were found in those aged older than 80 years (79% and 31%, respectively).

### Changes in PP levels

A significant reduction in PP levels during the treatment was also observed in the different age groups. Mean PP reductions after 3 months of treatment were 24.0 ± 11.1, 25.6 ± 11.0, 26.4 ± 11.8, and 25.5 ± 8.4 mmHg in the 55–59, 60–69, 70–79, and 80 years and older age groups, respectively. A PP of < 60 mmHg was achieved by 82% of patients after 3 months of treatment.

### Tolerability

Indapamide/amlodipine SPC was well tolerated by patients in the ARBALET study. A total of 16 adverse events were reported in 13/2217 patients (0.59%). Serious adverse events were reported in three (0.14%) patients: one hospitalization for unstable angina, one planned surgical intervention for cataracts, and one installation of a pacemaker. Of the non-serious adverse events, the most frequent was leg edema (5 events). One serious adverse event (unstable angina) and six non-serious adverse events (4 cases of leg edema, 1 dizziness and 1 tachycardia) led to patient discontinuation from the study.

Two cases (0.32%) of treatment-related adverse events (swelling/edema of legs and feet) were reported in this post-hoc analysis. Neither event led to patient discontinuation from the study.

## Discussion

According to the latest 2020 International Society of Hypertension guidelines, ISH is the most common form of essential hypertension in the young but is also frequently found in middle-aged individuals and in the elderly, in whom it reflects stiffening of the large arteries with an increase in PP [10]. Several studies have shown that ISH is associated with a significant increase in the risk of adverse cardiovascular outcomes including CAD, cerebrovascular disease and heart failure [6, 11, 12].

Most international guidelines support a diuretic and CCB combination for the treatment of ISH. This is based on data from studies in which a diuretic and CCB combination in subpopulations of patients with ISH was shown to be an effective treatment option for reducing SBP and cardiovascular events with a favorable safety profile [13–16], including studies with indapamide and amlodipine.

Both indapamide and amlodipine are supported by robust data in relation to this recommendation. Beyond their efficacy in reducing high blood pressure and improving target-organ damage, indapamide and amlodipine also have separately demonstrated benefits in terms of morbidity and mortality (HYVET, PATS, PROGRESS, ADVANCE, ALLHAT, ASCOT-BPLA, VALUE, ACCOMPLISH, CAMELOT, PREVENT, Syst-EUR, STOP Hypertension-2, Syst-China trials) [14, 17–26] with a decrease in total and cardiovascular mortality, and a reduction in morbidities related to the brain, heart, and kidney, depending on the study.

Findings from the study performed by Ojii et al. suggest that in black patients in sub-Saharan Africa amlodipine plus either hydrochlorothiazide or perindopril was more effective than perindopril plus hydrochlorothiazide at lowering blood pressure at 6 months [27].

We have performed a post-hoc analysis of the subgroup of patients with ISH from the ARBALET trial. In this subanalysis the addition or replacement of existing antihypertensive therapy with a once-daily indapamide/amlodipine 1.5/5 mg or 1.5/10 mg SPC for 3 months was associated with statistically significant BP reductions ranging from − 28.9 to − 32.5 mmHg compared with baseline across all age categories. Efficacy and safety of treatment in the ISH subgroup were comparable to results observed in the main ARBALET trial population [8, 9]. Reductions in office BP levels were observed as early as 2 weeks and continued to decrease such that mean values were in line with BP targets recommended by the 2018 ESC/ESH guidelines for the management of arterial hypertension by the end of the study. The SBP target of < 140 mmHg was met by 43.1% of patients at 2 weeks and by 92.5% at 3 months and the target of < 130 mmHg by 12.0% of patients at 2 weeks and by 55.9% at 3 months. As a result of the use of a single-pill combination of indapamide and amlodipine, the use of different classes of concomitant antihypertensive drugs decreased during the study. The exception was beta-blockers, for which prescriptions in patients with ISH increased from 31 to 34% during the study.

The degree of BP reduction remains the main determinant of vascular risk reduction in both young and elderly patients [28, 29]. While the highest proportion of patients achieving both the < 140 mmHg and < 130 mmHg targets at 3 months was observed in those aged 55–59 years, all age groups benefited from the replacement or addition of the SPC to their treatment plan.

It should nevertheless be noted that, although in the vast majority of patients the SPC either replaced previous antihypertensive therapy (73.5%) or was initiated (13.7%), there was a small proportion of patients (12.8%) in whom the SPC was added to existing antihypertensive treatment and consequently this could have had some influence on the results of the whole analysed population.

The effect of indapamide/amlodipine treatment on SBP has been demonstrated in two single-arm, open-label studies. In the NATIVE study (mean age of total study population 51 years), indapamide SR was added to background antihypertensive therapy [30]. In the subgroup of patients who received indapamide and amlodipine, SBP was decreased by 33 mmHg compared with baseline. In the EFFICIENT study (mean age 52 years) the single pill combination of indapamide/amlodipine at a dose of 1.5 mg/5 mg for 45 days resulted in a decrease in SBP of 29 mmHg compared with baseline [31].

The populations in the above trials were middle-aged, but the benefits of treating ISH in the elderly are also well established as first demonstrated 30 years ago in SHEP (mean age—72 years), where active treatment with a diuretic with or without a beta-blocker reduced mean SBP by 12 mmHg more than placebo [32]. Those randomized to diuretic treatment had marked reductions in the rates of myocardial infarction (− 27%), heart failure (− 55%), and stroke (− 37%). This was followed by the Syst-EUR trial where antihypertensive drug treatment with a CCB plus ACE-inhibitor or diuretic reduced SBP by 10 mmHg compared with placebo with reductions in cardiovascular outcomes similar to those in SHEP [24].

Older individuals comprise a large proportion of the ISH patients and with an aging population this is only expected to increase. The results from the ARBALET ISH cohort are in line with results of studies conducted in older individuals with ISH. In the SHEP study, the mean reduction of SBP was 26 mmHg on therapy with a diuretic or beta-blocker [32] compared with − 32.5 mmHg in the ARBALET ISH subgroup aged 70–79 years. Another study in elderly hypertensive patients, around a quarter of whom had ISH, compared the efficacy of indapamide sustained-release 1.5 mg in reducing BP versus amlodipine 5 mg and hydrochlorothiazide 25 mg [33]. In the ISH subgroup, indapamide 1.5 mg tended to have greater efficacy than hydrochlorothiazide at reducing SBP (− 24.7 vs − 18.5 mmHg, respectively; equivalence *P* = 0.117), and similar results to amlodipine (− 23 mmHg, equivalence *P* < 0.001) [33]. In the Medical Research Council study, patients aged 65–74 years with systolic hypertension, with or without diastolic hypertension, were randomized to diuretic, beta blocker, or placebo. SBP and DBP decreased in all groups, with the greatest systolic fall seen in the diuretic group in the first 3 months [34]. Considering the reduction in renin–angiotensin–aldosterone system (RAAS) activity with age and the prevalence of sodium-volume-dependent forms of hypertension in elderly patients, the use of an amlodipine/thiazide-like diuretic SPC represents a suitable option when choosing an antihypertensive therapy regimen in older patients.

SBP and PP are closely related and their elevated values are recognized as independent cardiovascular risk factors. In the ARBALET population with ISH, the addition of indapamide/amlodipine was associated with a significant reduction in PP which was reduced to below guideline recommended target (< 60 mmHg) in all age groups at 3 months. However, in some cases DBP, and consequently also PP, can be impacted by treatment. It was not the case in the current study: mean DBP reductions over 3 months were in the range of 6 mmHg compared with a mean SBP reduction of 32 mmHg.

The population of patients included in the present analysis of the ARBALET study comprised individuals aged 55 years and older with ISH, almost two thirds of whom were women, with a high prevalence of cardiovascular risk factors. The high rates of SBP control confirm data from previous studies showing that indapamide/amlodipine SPCas either a replacement or addition to existing therapy, or as preliminary therapy in treatment-naïve patients, may represent an effective means of reducing SBP in a broad range of patients of all ages, with common cardiovascular comorbidities.

Indapamide/amlodipine was well tolerated in the ISH subgroup with similar rates of adverse events to patients in the main ARBALET study. The adverse event profile was in line with the proven tolerability of indapamide and amlodipine, both alone and in combination.

### Study limitations

This was a post-hoc analysis of an observational open-label study without a control group and as such the results require confirmation in further clinical trials. Methods of routine clinical practice were used, including the auscultatory method for measuring BP. The SPC was added to existing antihypertensive therapy in 13% of patients and consequently this could have had some influence on the results of the whole analysed population. Another limitation is that the distribution of the indapamide/amlodipine doses in the different age groups was not analysed. Finally, this post-hoc analysis included only office BP measurements, while the main ARBALET study included both office and home BP measurements. HBPM data will be a subject for future analysis.

## Conclusion

In this post-hoc analysis of patients from the ARBALET trial with ISH, the single-pill combination of indapamide/amlodipine was associated with significant reductions in SBP in a broad range of patients of all ages typically found in clinical practice. Treatment was well tolerated and effective either when added to or replacing existing antihypertensive treatment, as well as in treatment-naïve patients, and was associated with high rates of target SBP and PP achievement.

## Data Availability

The datasets used and/or analyzed during the current study are available from the corresponding author on reasonable request.

## Abbreviations

ACE: Angiotensin-converting enzyme
BP: Blood pressure
CHF: Chronic heart failure
CVD: Cardiovascular disease
DBP: Diastolic blood pressure
HR: Heart rate
HT: Hypertension
ISH: Isolated systolic hypertension
PP: Pulse pressure
SBP: Systolic blood pressure
